# Efficient system for upstream mRNA trans-splicing to generate covalent, head-to-tail, protein multimers

**DOI:** 10.1038/s41598-018-36684-7

**Published:** 2019-02-19

**Authors:** Hiroaki Mitsuhashi, Sachiko Homma, Mary Lou Beermann, Satoshi Ishimaru, Hayato Takeda, Bryant K. Yu, Kevin Liu, Swetha Duraiswamy, Frederick M. Boyce, Jeffrey Boone Miller

**Affiliations:** 1 Department of Applied, Biochemistry School of Engineering, Tokai University Kanagawa, Yokohama, 259–1207 Japan; 2 Department of Neurology, Boston University School of Medicine Boston, Massachusetts, 02118 USA; 3 Department of Neurology, Massachusetts General Hospital, Boston, Massachusetts, 02114 USA

## Abstract

We present a plasmid-based system in which upstream trans-splicing efficiently generates mRNAs that encode head-to-tail protein multimers. In this system, trans-splicing occurs between one of two downstream splice donors in the sequence encoding a C-terminal V5 epitope tag and an upstream splice acceptor in the 5′ region of the pCS2(+) host plasmid. Using deletion and fusion constructs of the DUX4 protein as an example, we found that this system produced trans-spliced mRNAs in which coding regions from independent transcripts were fused in phase such that covalent head-to-tail protein multimers were translated. For a cDNA of ~450 bp, about half of the expressed proteins were multimeric, with the efficiency of trans-splicing and extent of multimer expression decreasing as cDNA length increased. This system generated covalent heterodimeric proteins upon co-transfections of plasmids encoding separate proteins and did not require a long complementary binding domain to position mRNAs for trans-splicing. This plasmid-based trans-splicing system is adaptable to multiple gene delivery systems, and it presents new opportunities for investigating molecular mechanisms of trans-splicing, generating covalent protein multimers with novel functions within cells, and producing mRNAs encoding large proteins from split precursors.

## Introduction

Trans-splicing joins regions of independently transcribed pre-mRNAs into chimeric mRNAs that can encode novel proteins or regulatory RNAs^1^. Trans-splicing was first identified in the joining of leader sequences to trypanosome mRNAs^2,3^. Further work has identified potential trans-splicing events in multiple prokaryotes and eukaryotes^1^, though the significance and occurrence of trans-splicing in humans and other vertebrates remains under investigation^4^. With the use of reconstituted spliceosomes and plasmid-transcribed mRNAs, trans-splicing can occur in a cell-free system *in vitro*^5,6^.

Use of trans-splicing to inactivate deleterious gene products or to generate functional proteins has been investigated as a potential therapeutic strategy for multiple diseases. In particular, strategies have been developed which use engineered “pre-trans-splicing molecules” (PTMs) to promote specific trans-splicing events in host cells^7–18^. PTMs are engineered RNAs that include the exons to be spliced into the target mRNA and a splice acceptor site coupled with a binding domain, typically ≥50 nucleotides long, that is complementary to an intron in the target mRNA. The binding domain localizes the PTM to near the splice donor site in the target mRNA and thereby increases the likelihood of trans-splicing^9^. In a cell-free system, trans-splicing can occur even when the two RNAs do not share such sequence complementarity, though splicing is more efficient when a binding domain is included^5^.

In this study, we identified a plasmid-based system in which upstream trans-splicing occurs to generate mRNAs that encode head-to-tail protein multimers. In this system, trans-splicing joined an upstream splice acceptor in the 5′ region of mRNA transcribed from the pCS2(+)-V5 host plasmid to one of two downstream splice donors in the sequence encoding a C-terminal V5 epitope tag. We validated this system using deletion and fusion constructs of the double homeobox DUX4 protein^19^, as well as the full-length zebrafish dok7 protein. DUX4 is aberrantly expressed and pathogenic in facioscapulohumeral muscular dystrophy (FSHD)^20^. Using this system, we produced trans-spliced mRNAs in which coding regions from independent transcripts were fused in phase, thereby generating chimeric mRNAs that encoded covalent head-to-tail protein multimers. Dimers were most abundant, but trimers and tetramers were found for some constructs. For a cDNA of ~450 bp, >40% of the expressed proteins were multimeric, though the efficiency of trans-splicing and extent of multimer expression decreased as cDNA length increased. Trans-splicing occurred in this system without the type of complementary binding domain found in PTMs. This plasmid-based trans-splicing system presents new opportunities for investigating molecular mechanisms of trans-splicing, identifying novel functions of covalent protein multimers, and generating larger proteins from split precursors.

## Results

**Figure 1:**
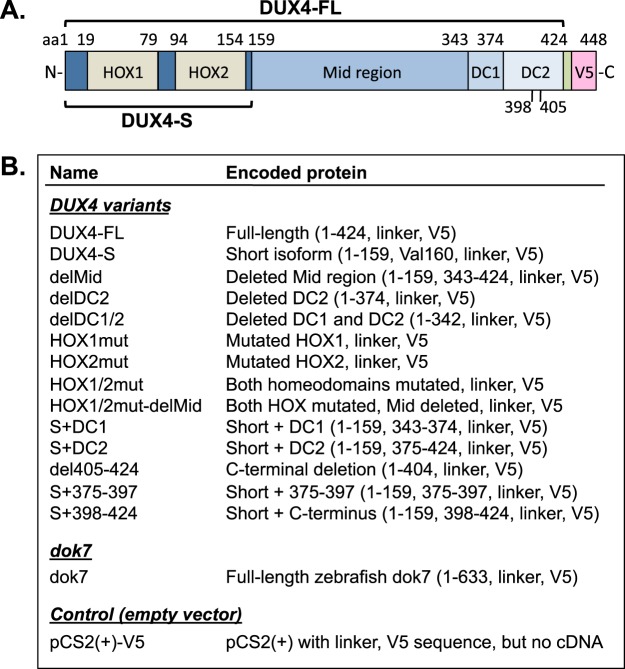
cDNA constructs that were cloned in the pCS2(+)-V5 plasmid. (**A**) Diagram of the DUX4-FL protein showing the two homeodomains (HOX1, HOX2), the mid-region, and the C-terminal domains (e.g., DC1 and DC2), as well as the linker (unlabeled green box) and the V5 epitope tag (V5) added to the C-terminus. The extents of the full-length (DUX4-FL, amino acids 1–424) and short (DUX4-S, amino acids 1–159) isoforms are indicated. (**B**) Table of cDNA constructs that were generated, cloned into the pCS2(+)-V5 host plasmid, and used in this study. Details of the mutations introduced into the HOX1 and HOX2 homeodomains to eliminate DNA binding were described previously^19^.

For our recent study of the functional domains of DUX4^19^, we generated a series of cDNAs for expression of DUX4 derivatives with deleted or mutated domains (Fig. 1). In brief, each of the cDNA constructs was cloned into the host plasmid pCS2(+)-V5^21^ with expression under control of a simian CMV IE94 promoter fragment^19^. The 3′ end of each construct was modified by addition of a sequence that encoded a seven amino acid linker (LEGTRFE) followed by the V5 epitope tag (GKPIPNPLLGLDSTRTG) and a stop codon (Fig. 1). The 3′UTR included a SV40 poly(A) signal sequence.

**Figure 2:**
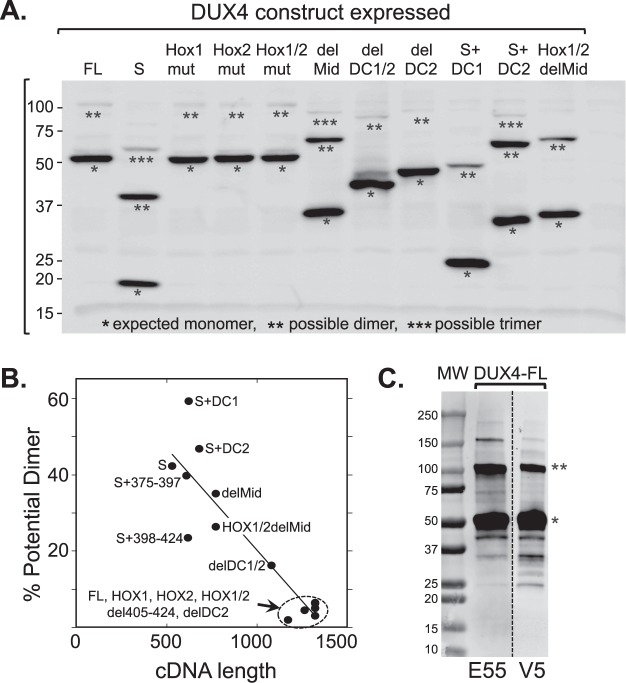
Expression of SDS-resistant, potential multimers upon transfection of DUX4 construct plasmids. (**A**) SDS-PAGE and immunoblotting were used to analyze proteins expressed in HeLa cells at 24 h after transfection with the indicated DUX4 pCS2(+)-V5 plasmids. On the upper blot, proteins were detected with a mAb that reacted with the V5 epitope on the C-terminus of each construct. In this figure and all following figures, each band marked with a single asterisk is the size predicted for a monomeric protein expressed from the corresponding cDNA construct, whereas bands marked with double and triple asterisks are the sizes expected for potential dimers and trimers, respectively. The potential dimer band was most noticeable for DUX4-S (lane 2). Numbers to left of blot indicate molecular mass in kDa. (**B**) Densitometry of immunoblots was used to determine the percentage of protein expressed from each construct that was the size of a potential dimer. Linear curve-fitting was used to derive the solid line (y = 73.6-0.053x, R = 0.90). The percentage of potential dimers decreased as the cDNA insert became longer. (**C**) In a separate experiment, HEK293 cells were transfected with the DUX4-FL plasmid for 48 h and the resulting proteins were detected by immunoblotting either with mAb E55, which is specific to an epitope in the C-terminal portion of DUX4-FL^22^, or with anti-V5 as indicated. Two major bands reacted with both antibodies, one the size of the expected monomer (single asterisk) and a second the size of a potential dimer (double asterisk). Also seen were potential higher order multimers, as well as intermediate and smaller bands that could be proteolytic products. This result shows that the SDS-resistant, potential multimers included the E55 (endogenous DUX4-FL) and V5 (tag) epitopes. MW = markers of protein molecular mass shown in kDa.

When we transfected HeLa or HEK293 cells with individual DUX4 pCS2(+)-V5 plasmids and analyzed the expressed proteins by SDS-PAGE and immunoblotting for the V5 epitope, we found that, in addition to bands of the size predicted for monomers, there also were bands that were the size of potential dimers and, in some cases, of even higher order multimers (Fig. 2A and ref.^19^). In HeLa cells transfected with the DUX4 pCS2(+)-V5 plasmids, bands of the sizes expected for potential dimers or higher order multimers appeared to be most abundant for the smallest of the expressed proteins, e.g., the DUX4-S monomer at ~20 kDa; and the percentage of possible dimers appeared to be roughly inversely proportional to the size of the cDNA and the expressed protein (Fig. 2B). When DUX4-FL was expressed in HEK293 cells, we found that mAb E55 which is specific for an epitope on the C-terminal portion of DUX4-FL^22^, as well as the anti-V5 mAb, detected both the expected monomer (~50 kDa) and the potential dimer (~100 kDa) bands (Fig. 2C). Thus, the apparent multimeric protein contained both the DUX4-FL-specific E55 epitope and the V5 epitope. In further studies we characterized these multimers as covalent proteins and identified key aspects of the plasmids that were required for multimer formation (see Supplementary Data, including Supplementary Figs S1 and S2).

When considering the possible nature of the multimers generated from the pCS2(+)-V5 plasmids, we noted the work of Ansseau *et al*.^23^ who identified two sites within the V5 epitope DNA sequence that could function as apparently cis-splice donors when an intron-containing sequence was located downstream (3′) of the C-terminal V5 epitope tag. Though the pCS2(+)-V5 DNA constructs that we used contained the same V5 epitope sequence analyzed by Ansseau *et al*.^23^ the 3′UTRs of our constructs contained only the intronless SV40 (polyA) sequence which Ansseau*et al*.^23^ noted would not generate aberrant downstream splicing.

**Figure 3:**
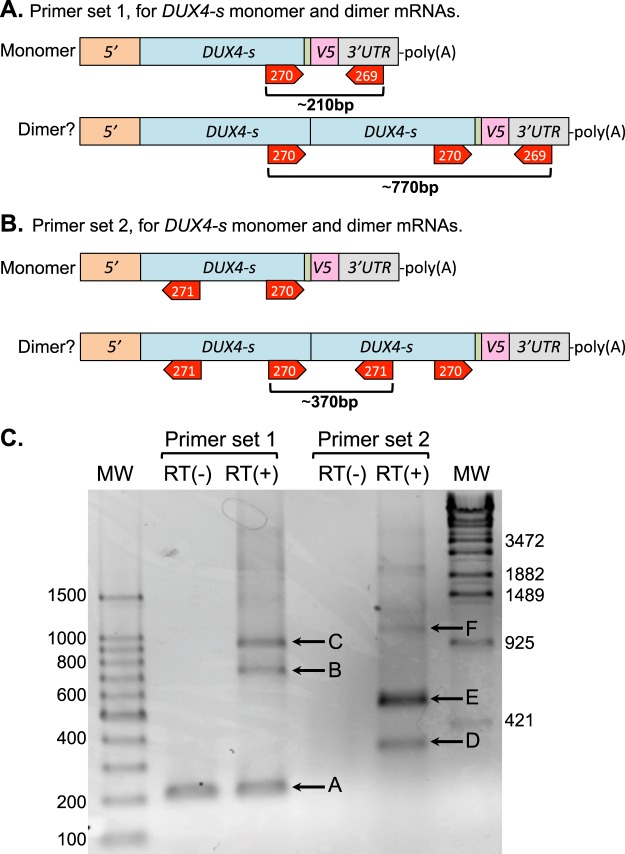
Detection of possible trans-spliced mRNAs. (**A**) Strategy to detect potential trans-splicing sites in DUX4-S-V5. Primer set 1, which consisted of forward primer #270 and reverse primer #269, was designed to amplify products predicted to be of ~770 bp from a trans-spliced mRNA and ~210 bp product from an mRNA encoding the expected monomer. (**B**) An alternate strategy using Primer set 2, which consisted of forward primer #270 and reverse primer #271, was designed to amplify an ~370 bp product from a trans-spliced mRNA but no product from the mRNA encoding a monomer. (**C**) Detection of possible trans-spliced mRNAs. HeLa cells were transfected with DUX4-S-V5 for 24 h and transcription products were analyzed by PCR with primer sets 1 and 2, without (−RT) or with (+RT) reverse transcriptase. Under primer set 1, the band labeled “A” in the −RT lane arose from unremoved plasmid cDNA, but the bands labeled “B” and “C” were RT-dependent and potentially due to spliced mRNAs. Under primer set 2, the RT-dependent bands labeled “D,” “E,” and “F” were potentially due to spliced mRNAs. See Fig. 4 and text for analyses of the labeled bands.

Though downstream splicing within a single mRNA thus seemed unlikely, we hypothesized that the apparent dimers could have been generated if a downstream splice donor (i.e., in the V5 epitope) on one mRNA was joined to an upstream splice acceptor on a second mRNA to generate a chimeric mRNA that encoded a covalent, head-to-tail dimer (or multimer if splice events were repeated). To test this possibility, we designed PCR-based strategies that would allow us to identify trans-spliced mRNAs that encoded such dimers using DUX4-S as a test (Fig. 3A,B).

The first strategy to detect trans-spliced mRNAs used primer set #1 (Fig. 3A) and produced three major PCR products (Fig. 3C). Band “A” at ~210 bp was the size expected for the *DUX4-s* monomer, but this band was independent of reverse transcriptase so likely arose from unremoved plasmid DNA in addition to monomer mRNA. In contrast, the two larger products, band “B” at ~750 bp and band “C” at ~900 bp (Fig. 3C), were both reverse transcriptase-dependent and large enough to have been produced from a trans-spliced mRNA. Accordingly, we cloned the PCR products into the pCR blunt vector and sequenced bands “B” and “C.”

**Figure 4:**
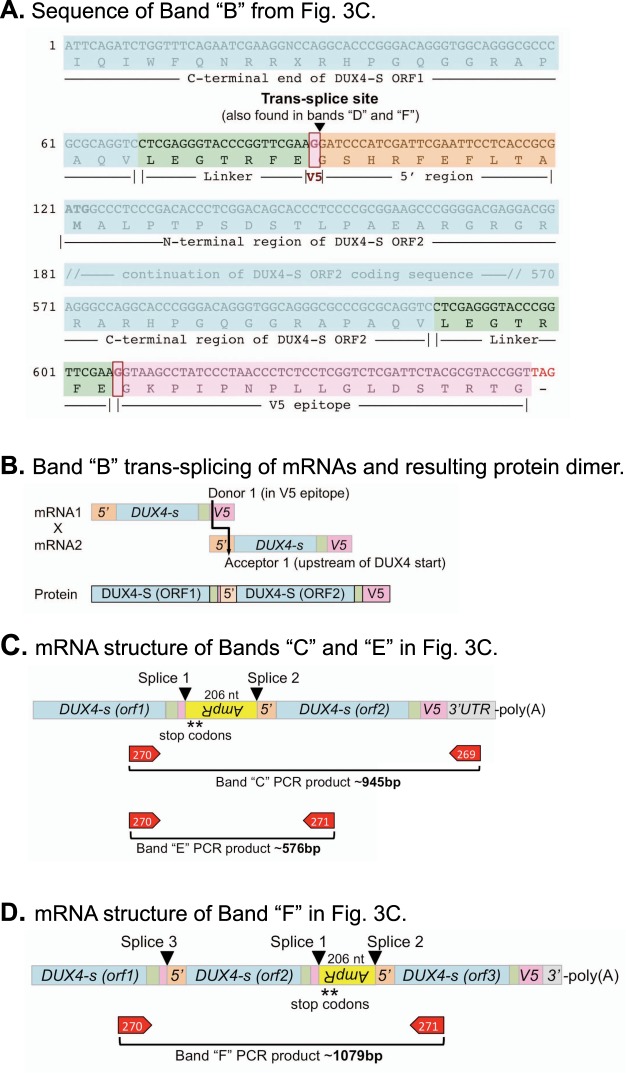
Identification of trans-spliced mRNAs produced from pCS2(+)-DUX4-S-V5. (**A**) Sequence of PCR band “B” from Fig. 3C determined with reverse primer #269 included (in order from 5′ to 3′): (i) the C-terminal portion of a DUX4-S open reading frames (DUX4-S ORF1, light blue box); (ii) the first linker sequence (light green box); (iii) a single G nucleotide from the V5 epitope (light red box) which, at the indicated trans-splicing site, was joined to; (iv) a sequence from the 5′ region just upstream of DUX4-S cDNA in the original plasmid (light orange box); (v) a second complete DUX4-S (ORF2, light blue box, initial ATG in bold); (vi) a second linker (light green box); (vii) a complete V5 epitope sequence (light red box); and (viii) a TAG stop codon (shown in red). The indicated trans-splicing site and surrounding sequences were also found in bands “D” and “F” in Fig. 3C. (**B**) The upper diagram shows how “Band B” was generated by trans-splicing of two mRNAs (mRNA1 x mRNA2) using a donor site at the beginning of the V5 epitope and an acceptor site in the 5′ region upstream of the DUX4-S cDNA. The lower diagram shows the covalent, head-to-tail, DUX4-S-V5 dimer that would be generated from the trans-spiced mRNA. (**C**) Diagram structures of PCR bands “C” and “E” in Fig. 3C as determined from sequencing. In this case, two splicing events occurred. One splice (Splice 1) joined the donor site in the V5 epitope (as also used in band “B”) to an acceptor site in the downstream ampicillin resistance (*AmpR*) gene. This first splice could have arisen either by splicing within a single mRNA or by trans-splicing. The second splice (Splice 2) joined a donor site in *AmpR* to the acceptor site (as also used in band “B”) in the 5′ region upstream of the DUX4-S cDNA and thus must have arisen by trans-splicing. The 206 nucleotide (nt) sequence of the *AmpR* gene was in the reverse coding orientation (as indicated by the upside down and reversed *AmpR* symbol in the yellow box) and contained two stop codons in the DUX4-S reading frame as indicated by the asterisks. As seen in the diagram, the only intact V5 epitope sequence in this mRNA was downstream (3′) of the stop codons in *AmpR*, so no V5-tagged protein would have been produced from this mRNA. (**D**) Diagram structure of PCR band “F” in Fig. 3C as determined from sequencing. In this case, three splicing events occurred. The sites labeled Splice 1 and Splice 2 were the same as in panel C. The site labeled Splice 3 resulted in the inclusion of a third *DUX4-s* open reading frame in the predicted mRNA.

The sequence of band “B” showed that it was indeed derived from a trans-spliced mRNA (Fig. 4A). In this case, a donor site at the beginning of the V5 epitope sequence in one mRNA was spliced to an acceptor site that was 5′ to the DUX4-S start site and was in a separate mRNA as diagramed in Fig. 4B. This donor site (which we termed Donor 1 or D1) was the same as the cis-splicing donor site identified by Ansseau *et al*.^23^, but the acceptor site (A1) and the occurrence of upstream trans-splicing had not been noted previously. Translation of this trans-spliced mRNA would produce a covalent, head-to-tail, DUX4-S dimer as diagramed in Fig. 4B. Note that trans-splicing removed the V5 epitope from the first DUX4-S open reading frame (ORF), but left the V5 epitope on the second DUX4-S ORF.

The second trans-spliced mRNA identification strategy used primer set #2 (Fig. 3B) and produced three major reverse transcriptase-dependent PCR products (Bands “D,” “E,” and “F” in Fig. 3C). Band “D” at ~370 bp was the size expected for the *DUX4-s* dimer, and direct sequencing identified the same trans-splice site in this band as was found in band “B” (Fig. 4A and not shown).

Sequencing of bands “C,” “E,” and “F” produced unexpected results: these bands were produced from multiple separate splicing events (Fig. 4C,D). Bands “C” and “E” were amplified from mRNA with identical sequences by the two different primer sets. In this mRNA, there were two splices. One splice occurred when the donor 1 site in the V5 epitope was joined to a previously unrecognized downstream acceptor site located in the Ampicillin resistance (*AmpR*) gene (labeled Splice 1 in Fig. 4C). The second splice (labeled Splice 2) used a donor site, also previously unrecognized, in the *AmpR* gene that was joined to the upstream, acceptor 1 site that we also identified in bands “B” and “D” (Fig. 4A,B). The resulting trans-spliced mRNA contained two *DUX4-s* ORFs separated by 206 nucleotides of the reversed orientation *AmpR* coding sequence. This mRNA would not have produced a V5-tagged protein product because the reversed orientation of the AmpR sequence included two stop codons and the V5 epitope was removed by trans-splicing from the first *DUX4-s* open reading frame (Fig. 4C). Splice 1 in band “E” could have arisen either by splicing within a single mRNA or by trans-splicing, but splice 2, which coupled a downstream donor to an upstream acceptor, must have arisen by trans-splicing.

Sequencing of band “F” which was a PCR product amplified by primer set 2 identified an mRNA that contained a third splice (labeled Spice 3 in Fig. 4D) in addition to the two splices found in bands “C” and “E.” This third splice led to the inclusion of a third DUX4-s open reading frame in the transcript (Fig. 4D). The protein produced from this transcript would have been dimeric but would also have lacked a V5 epitope.

**Figure 5:**
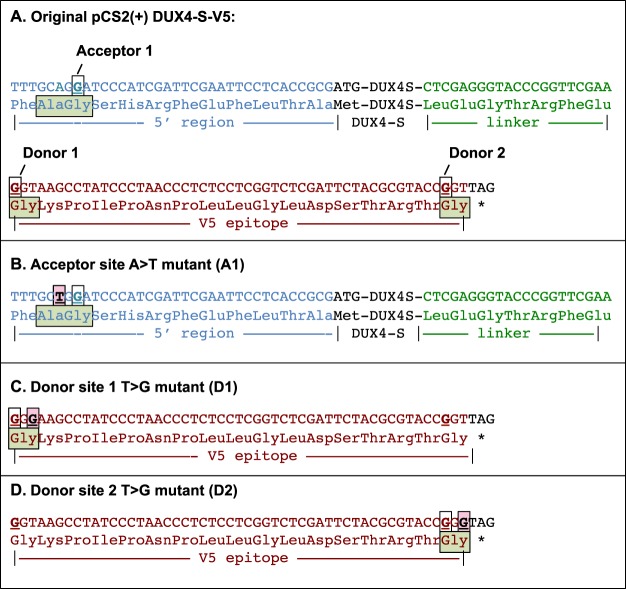
Acceptor and donor splice sites in the pCS2(+)-based DUX4-S-V5 plasmid and generation of mutant plasmids used to test functionality of these sites. (**A**) Based on sequencing of bands “B,” “D,” and “F” in Fig. 3C, we identified, as indicated, an acceptor site (blue G, boxed and underlined) in the 5’ region upstream of the DUX4-S cDNA. This acceptor sequence had a NetGene2 acceptor prediction confidence = 0.71. The sequencing also identified a first donor site (Donor 1, brown G, boxed and underlined), which had a NetGene2 splice donor site prediction confidence = 0.79. A second possible donor site (Donor 2, brown G, boxed and underlined) was located at the downstream (3′) end of the V5 epitope sequence as noted by Ansseau *et al*.^23^. This site had a NetGene2 splice donor site prediction confidence = 0.62. The amino acid(s) encoded near the acceptor and donor splice sites are shown in light green boxes. (**B**) To disable the acceptor site, we produced the A1 mutant by making an A to T switch of the nucleotide outlined by the light red box in the 5′ region upstream of the DUX4-S cDNA. (**C**) To disable the donor 1 site, we produced the D1 mutant by making a T to G switch of the nucleotide outlined by the light red box near the 5′ end of the V5 epitope. (**D**) To disable the donor 2 site, we produced the D2 mutant by making a T to G switch of the nucleotide outlined by the light red box at the 3′ end of the V5 epitope. Note that the encoded amino acid sequence was not altered by any of the three mutations. In addition, NetGene2 analyses predicted that none of the mutated sequences would function as acceptors or donors.

**Figure 6:**
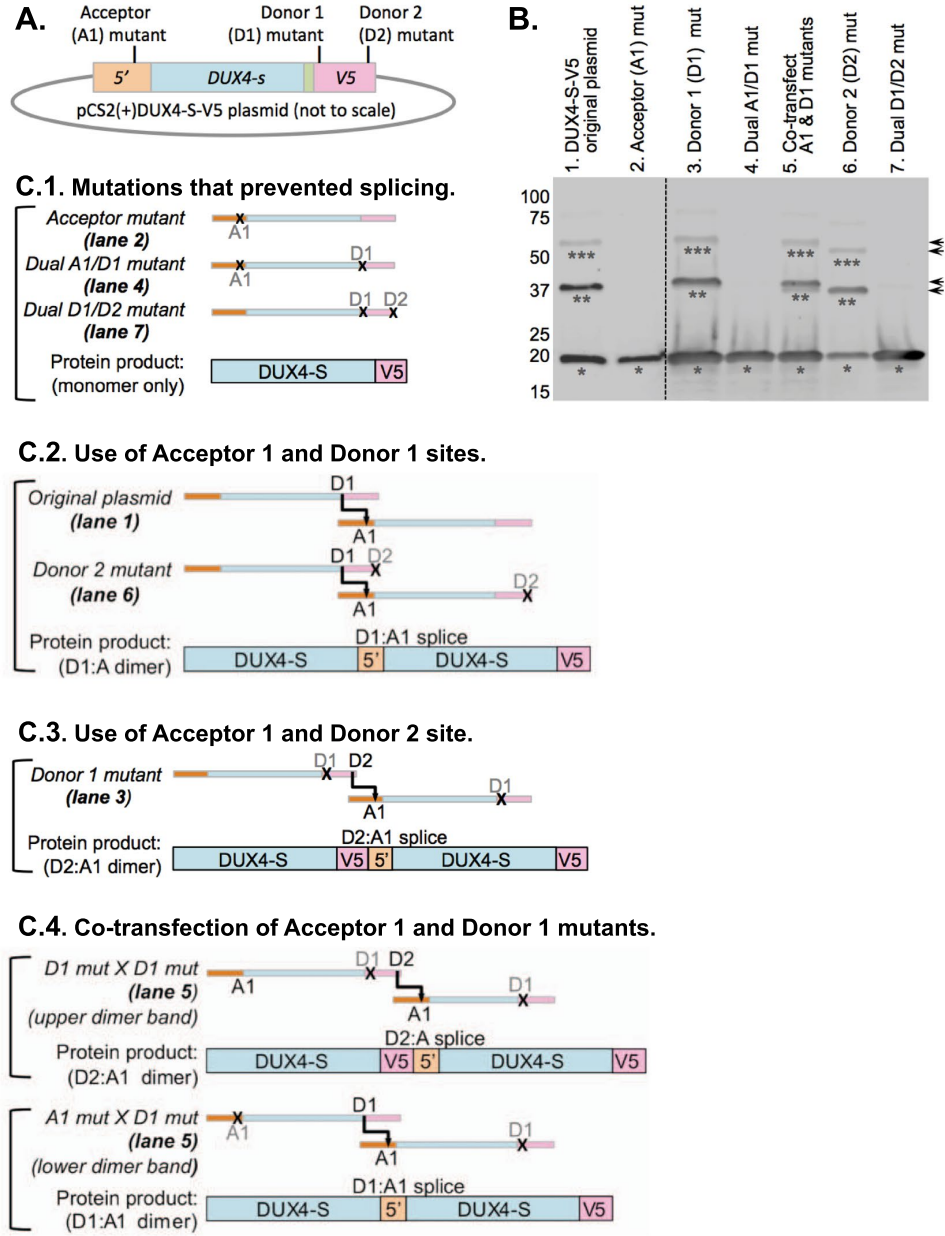
Confirmation of acceptor and donor splice sites. (**A**) Diagram of DUX4-S-V5 plasmid showing approximate locations of acceptor (A1) and donor site (D1, D2) mutations (see Fig. 5 for details). (**B**) Immunoblot of V5-tagged proteins produced 48 h after transfection of HEK293 cells. Apparent monomers, dimers, and trimers are indicated by single, double, and triple asterisks respectively. No apparent multimers were generated when a single plasmid was transfected in which either (i) the acceptor site (lane 2), (ii) both the acceptor and donor 1 sites (lane 4), or (iii) both donor sites (lane 7) were mutated. Multimers were generated, however, when only one of the donor sites was mutated (lane 3, D1; and lane 6; D2), but the multimers generated when the D1 site was mutated were larger in size (arrows) as expected if the D2 site was used when the D1 site was disabled. When two separate plasmids were co-transfected (lane 5), one with the acceptor mutation and one with the donor 1 mutation, multimers were formed as expected for trans-splicing. (**C**) Diagram of trans-splicing patterns underlying the immunoblot results in panel B. In each diagram, the mRNAs are pictured schematically with the 5′ region in light orange, the DUX4-S coding sequence in light blue, and the V5 epitope in light red. Functional acceptor (A1) and donor (D1, D2) sites are indicated with black lettering, whereas mutated sites are indicated with gray lettering and an “X.” For each diagram, the corresponding lane of the immunoblot in panel B is indicated. Note that for lane 5 (C.4, Co-transfection of Acceptor 1 and Donor 1 mutants), two dimers of different sizes are possible and, indeed, two dimer bands are seen in lane 5 of panel B, though the smaller band is fainter than the larger band.

To confirm that the donor and acceptor splice sites we identified by sequencing were functional, we next mutagenized the sites in the pCS2(+)-DUX4-S-V5 plasmid and determined how mutagenesis affected generation of multimeric proteins (Figs 5 and 6). Using the NetGene2 splice site prediction algorithm^24,25^, we designed a mutagenesis strategy to disrupt the splice sites while maintaining the original amino acid coding sequence (Fig. 5). We generated mutated plasmids in which we disabled (i) the acceptor site 5′ to the DUX4-S cDNA (designated A1), (ii) the first donor site in the V5 epitope (D1), (iii) second predicted donor site in the V5 epitope (D2), and (iv) both acceptor 1 and donor 1 sites (A1/D1), and (v) both donor sites (D1/D2). These mutants are shown in detail in Fig. 5 and are diagramed in Fig. 6A. We transfected the original plasmid and each of the mutant plasmids into HEK293 cells and, after 48 h, used immunoblotting with anti-V5 mAb to determine if multimers were generated.

As shown in Fig. 6B, we found that multimers were not generated from three of the mutant plasmids: A1 (lane 2), A1/D1 (lane 4), and D1/D2 (lane 7). Thus, as diagramed in Fig. 8C.1, multimer formation was prevented either when the acceptor site was mutated (in the A1 and A1/D1 plasmids) or when both of the potential donor sites were mutated (D1/D2 plasmid). When only the second donor site was mutated, however, the pattern of multimer formation was the same as produced by the unmutated pCS2(+)-DUX4-S-V5 plasmid (Fig. 6B, compare plasmid D2 in lane 6 with original plasmid in lane 1). Thus, as diagramed in Fig. 6C.2, the original and D2 plasmids both appeared to use the same acceptor (A1) and donor (D1) sites for trans-splicing. Because our previous study of DUX4 function^19^ used pCS2(+)-V5 plasmids that generated multimeric proteins, we repeated assays from our previous studies using the pCS2(+)-DUX4-S-V5 plasmids with the A1 and D1/D2 mutations to prevent multimer formation. We found that the mutant plasmids produced the same results, indicating that the DUX4-S multimers did not affect the outcomes of the functional assays (e.g., competition between DUX4-S and DUX4-FL for promoter binding) used in our earlier study (not shown).

Though multimers were also generated when only the first donor (D1) site was mutated, the dimer and trimer bands generated from the D1 mutant plasmid were slightly larger than the bands produced from the original or D2 mutant plasmid (Fig. 6B, compare plasmid D1 in lane 3 with original plasmid in lane 1 and D2 plasmid in lane 6). Because no multimers were generated when both D1 and D2 were mutated, this result indicates that, although the D1 site in the V5 epitope was used preferentially, the D2 site was used when the D1 site was inactivated, as diagramed in Fig. 6C.3. When the D2 site was used, the dimers included ~16 additional amino acids from the V5 epitope, thus accounting for their larger size.

To further assess trans-splicing patterns and multimer formation, we co-transfected the A1 mutant and D1 mutant plasmids. In this case, multimers were formed, but two dimer bands were formed (Fig. 6B, lane 5). The larger, major band appeared to correspond to trans-splicing between two D1 mutant mRNAs and used the unmutated acceptor A1 site and the second donor site (D2). The smaller, minor band appeared to correspond to trans-splicing which used the unmutated acceptor site (A1) on the D1 mutant mRNA and the unmutated first donor site (D1) on the A1 mutant mRNA. These splicing patterns are diagramed in Fig. 6C.4.

**Figure 7:**
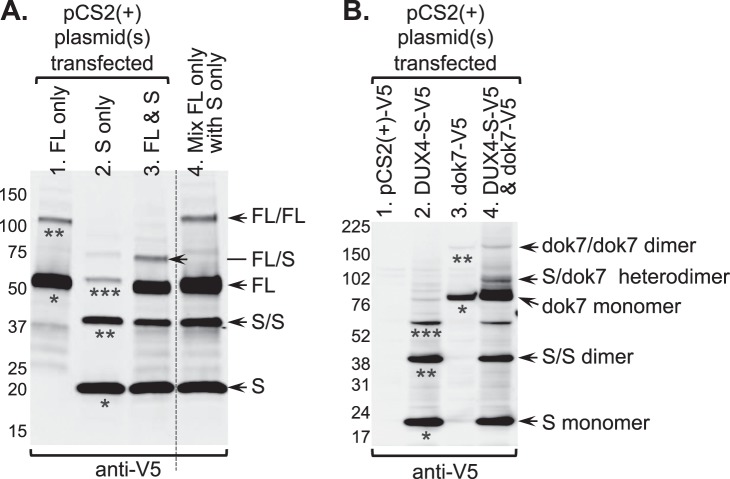
Generation of covalent heterodimeric proteins from trans-spliced mRNAs. (**A**) Heterodimer of DUX4-FL and DUX4-S generated upon co-transfection of plasmids based on pCS2(+)-V5. HEK293 cells were transfected with 5 µg DUX4-FL-V5 (lane 1, FL only), 5 µg DUX4-S-V5 (lane 2, S only), or co-transfected with 2.5 µg each of DUX4-FL-V5 and DUX4-S-V5 (lane 3, FL & S), all in the pCS2(+)-V5 plasmid. After 48 h, epitope-tagged proteins (20 µg/lane) were analyzed by SDS-PAGE and anti-V5 immunoblotting. For lane 4, the cell extracts from the single transfection were simply mixed together prior to SDS-PAGE. The DUX4-FL-V5 plasmid generated bands the size expected for both a monomer (single asterisk in lane 1, also indicated by arrow labeled FL to right of blot) and a dimer (double asterisk in lane 1, arrow labeled FL/FL). Similarly, the DUX4-S-V5 plasmid generated bands the size expected for a monomer (single asterisk in lane 2, arrow labeled S), a dimer (double asterisk in lane 2, arrow labeled S/S), and possible trimer (triple asterisk in lane 2). When the DUX4-FL-V5 and DUX4-S-V5 plasmids were co-transfected, the band at the size of the potential DUX4-FL-V5 dimer (FL/FL) diminished in intensity and a new band appeared at the size expected of a potential DUX4-FL/DUX4-S heterodimer (indicated by arrow labeled FL/S). The potential heterodimer band was not found when the single cell extracts were mixed (lane 4). (**B**) Heterodimer of DUX4-S and zebrafish dok7 generated upon co-transfection. HeLa cells were transfected with: 1 µg of empty control pCS2(+)-V5 plasmid (lane 1); 1 µg DUX4-S-V5 plasmid (lane 2); or 1 µg dok7-V5 plasmid (lane 3); or co-transfected with 0.5 µg each of DUX4-S-V5 and dok7-V5 plasmids (lane 4). After 48 h, epitope-tagged proteins (20 µg/lane) were analyzed by SDS-PAGE and anti-V5 immunoblotting. As previously, the DUX4-S-V5 plasmid generated monomers (single asterisk in lane 2 and arrow labeled S to right of blot), dimers (double asterisk in lane 2, and arrow labeled S/S) and trimers (triple asterisk). The dok7-V5 plasmid generated a strong band the size expected for a monomer (~80 kDa, single asterisk in lane 3, arrow labeled dok7 monomer), as well as a fainter band the size expected for a dimer (~160 kDa, double asterisk in lane 3, arrow labeled dok7/dok7 dimer). When the DUX4-S-V5 and dok7-V5 plasmids were co-transfected, a new band appeared at the size expected of a potential DUX4-S/dok7 heterodimer (~100 kDa, indicated by arrow labeled S/dok7 heterodimer).

Finally, we used co-transfection experiments to test our expectation that covalent, heterodimeric proteins could be produced by trans-splicing of mRNAs that encode different proteins. In one set of experiments, we co-transfected pCS2(+)-DUX4-FL-V5 and -DUX4-S-V5 plasmids and found that a band of the size expected for a DUX4-FL/DUX4-S heterodimer was produced (Fig. 7A, lane 3, indicated by FL/S arrow). This band was not found upon transfection of only the DUX4-FL-V5 (Fig. 7A, lane 1) or DUX4-S-V5 (Fig. 7A, lane 2) plasmids, or when cell extracts from the single transfections were simply mixed together prior to SDS-PAGE (Fig. 7A, lane 4). In the co-transfection, the amount of DUX4-FL dimer was much reduced (compare FL/FL bands in lane 1 vs. lane 3) as the FL/S heterodimer was formed. This result indicates the *DUX4-fl* mRNA was preferentially trans-spliced to the shorter *DUX4-s* mRNA rather to another one of the longer *DUX4-fl* mRNAs.

**Figure 8:**
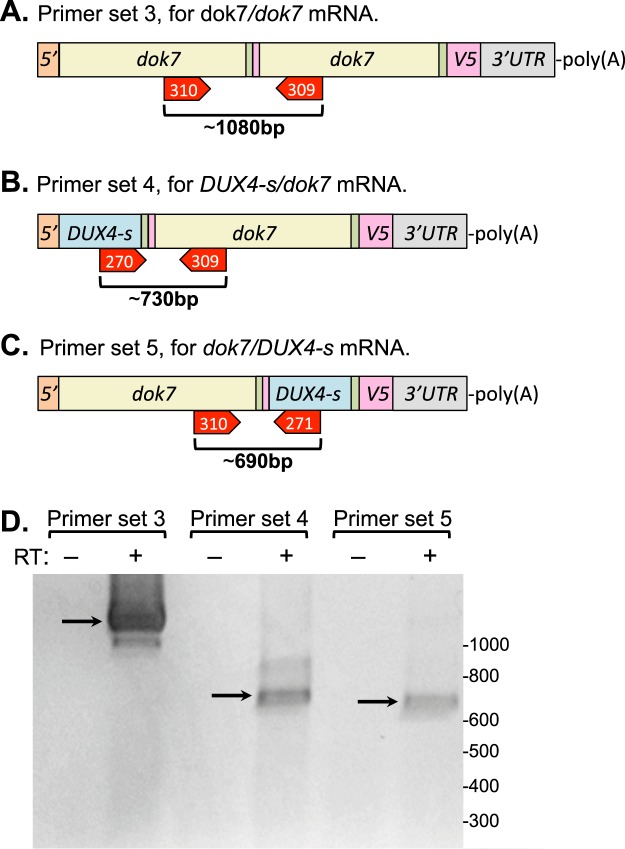
Identification of mRNAs generated by trans-splicing of DUX4-s and dok7 mRNAs. (**A**) Diagram of primer set 3 (primers #310 and #309) designed to generate a RT-PCR product of ~1080 bp from trans-spliced mRNA containing two sequential *dok7* coding sequences. (**B**) Diagram of primer set 4 (primers #270 and #309) designed to generate a RT-PCR product of ~730 bp from trans-spliced mRNA in which the *DUX4-s* coding sequence is upstream of (5′ to) the *dok7* coding sequence. (**C**) Diagram of primer set 5 (primers #310 and #217) used to identify an alternative configuration of the trans-spliced mRNA in which *dok7* is upstream of (5′to) *DUX4-s*. (**D**) All three sets of primers generated reverse transcriptase (RT) dependent bands of the expected sizes (arrows), indicating that all forms of the trans-spliced mRNAs were generated. Sequencing of the bands indicated by arrows confirmed that trans-splicing joined the donor 1 site in the V5 epitope sequence of one mRNA to the upstream acceptor 1 site in the 5′ region of the second mRNA, exactly as seen above for trans-splicing of two *DUX4-s* mRNAs, i.e., as in band “B” of Figs 5 and 6A,B.

In a second set of experiments, we co-transfected the pCS2(+)-DUX4-S-V5 plasmid with a pCS2(+)-*dok7*-V5 plasmid (Fig. 1B) for expression of zebrafish *dok7*. In this co-transfection, we found that, in comparison to the single transfections, an additional band of the size expected for a DUX4-S/dok7 chimeric protein was produced (Fig. 7B, lane 4, indicated by S/dok7 heterodimer arrow). In this experiment, the dok7/dok7 dimer did not appear to be reduced in the co-transfection compared to the single transfection (Fig. 7B, compare lanes 3 and 4). We then designed sets of PCR primers to distinguish between *dok7/dok7* homodimeric mRNA and two possible chimeric mRNAs, *DUX4-s/dok7* and *dok7/DUX4-s*, distinguished by the 5′ to 3′ order in which the *DUX4-s* and *dok7* coding sequences appear (Fig. 8A–C). By RT-PCR, we found that all three of these potential trans-spliced mRNAs were generated upon co-transfection (Fig. 8D). Sequencing of the RT-PCR products confirmed that the trans-spliced *dok7/dok7*, *DUX4-s/dok7*, and *dok7/DUX4-s* mRNAs were all generated using the same upstream Acceptor 1 and downstream Donor 1 splice sites described above (Figs 5 and 6). The DUX4-S/DUX4-S homodimer was also generated (Fig. 7B). This experiment confirmed that RNAs transcribed from two different plasmids were trans-spliced and that mRNAs with the two coding sequences in different 5′ to 3′ order could be generated in different upstream trans-splicing events.

Finally, we used online prediction tools to analyze splice site strength and for the presence of splicing enhancers or suppressors (see Supplementary Data for details). Based on these analyses, the acceptor (A1) and donor (D1, D2) splice sites were predicted to be the strongest splice sites in the DUX4-S-V5 and dok7-V5 mRNAs transcribed from the pCS2(+) vector (Supplemental Fig. S3). The acceptor and donor sites that we identified in the ampicillin resistance gene were also predicted to be very strong sites (Supplemental Fig. S3). In addition, a complex landscape of possible splicing enhancers and suppressors was predicted in the DUX4-S-V5 mRNA (Supplemental Fig. S4).

## Discussion

In this work, we identified a plasmid-based system in which mRNA trans-splicing occurred via joining a downstream splice donor to an upstream splice acceptor. This process of upstream trans-splicing generated chimeric mRNAs that encoded covalent, head-to-tail protein multimers. Dimers were the most abundant multimer, but higher order multimers (trimers, tetramers) were found in some cases (e.g. DUX4-S lanes in Figs 2A and 4B). Two general conclusions arise from this study. First, our study provides a cautionary example of the potential for unexpected upstream trans-splicing events in mRNAs generated from a commonly used plasmid and epitope tag. Second, this plasmid-based trans-splicing system presents new opportunities for investigating molecular mechanisms of trans-splicing, identifying novel functions of covalent protein multimers, and generating larger proteins from split precursors.

As noted by Ansseau *et al*.^23^ caution must be used when working with plasmids containing functional donor sites in the V5 epitope sequence due to cis-splicing when downstream intron/exon sequences are present. Our work further emphasizes and extends this caution by additionally showing that the V5 splice donor sequences can join with suitable acceptor sites either upstream via trans-splicing or as far downstream as the ampicillin resistance gene (~1500 nucleotides from the V5 sequence). The unexpected splice acceptor and donor sites that we identified were all predicted with high confidence by the NeuralNet2 algorithm. We and Ansseau *et al*.^23^ showed that the V5 splice donor sites could be inactivated by mutation while maintaining the V5 amino acid sequence, and we similarly showed that the upstream acceptor site (A1) could be similarly inactivated by mutation while maintaining the encoded amino acid sequence.

The two splice donors (which we termed D1 and D2) located in the V5 epitope sequence were previously identified by Ansseau *et al*.^23^ who found that these donor sites participate in downstream cis-splicing when introns and exons are located downstream of the V5 sequence. Trans-splicing was not noted by Ansseau *et al*.^23^ In our constructs, we used the intronless SV40 (polyA) sequence, which would not have been expected to generate aberrant splicing^23^, downstream of the V5 epitope. However, in addition to upstream trans-splicing, we unexpectedly found an example of downstream splicing in which 256 nucleotides of the ampicillin-resistance coding sequence were included, in reverse orientation, in a chimeric mRNA that appeared to have been generated by two separate splices including one with the V5 donor site (Fig. 4C). Further work is needed to determine if this chimeric mRNA was generated by two trans-splices or by a combination of a cis- and a trans-splice. Further work is also needed to understand the origin of the RNA that included the ampicillin resistance sequence. A likely possibility is that the sequence was included in a long, unprocessed RNA generated from the usual CMV promoter transcription start site and that this nascent RNA was spliced before poly(A) addition occurred. A less likely alternative is that the ampicillin resistance sequence may have been included in an RNA produced from an alternative, as yet unidentified, transcription start site.

Several features of the pCS2(+)-V5 host plasmid allowed trans-splicing and production of protein multimers. In particular, there was an open reading frame in the ~30 nucleotides between the upstream splice acceptor site (A1) and the translation start site, and this ORF was in phase with the inserted cDNAs. Second, the trans-splicing reaction maintained an open reading frame from the first DUX4 ORF though the splice site and into the second DUX4 ORF. Trans-splicing would likely still have occurred if a stop codon had been generated at the splice site, but no multimeric proteins would have been generated. Also, the transcription start site in the simian CMV IE94 promoter, as identified by Isomura *et al*.^26^, was located ~100 nucleotides upstream of the splice acceptor site. Thus, the nascent transcripts had more than 18–40 nucleotides upstream of the acceptor site as is necessary for splicing^9^.

In contrast to engineered PTMs that include a complementary binding domain (typically >50 nucleotides long) to guide trans-splicing, the plasmid-based system used here did not appear to require such a binding domain. First, the cDNA itself did not appear to affect trans-splicing as the RNAs for two unrelated sequences, *DUX4* and *dok7*, were both trans-spliced. Second, we did not find long complementary sequences that might position the acceptor and donor sites of different RNAs near each other — the longest complementary regions we found were only ≤5 nucleotides (Supplementary Fig. S5). Thus, though trans-splicing of viral SV40 mRNAs uses complementary regions of only 11–16 nucleotides^27^, the RNAs transcribed in the plasmid system did not have even this level of complementarity. Additional mutational and functional analyses are needed to determine if particular sequences outside the splicing sites might function, even with limited complementarity, to bring together donor and acceptor sites on different mRNAs, thereby promoting trans-splicing.

In the plasmid system described here, trans-splicing could occur with high efficiency despite the lack of a complementary binding domain. In particular, for the *DUX4-s* construct with a cDNA of ~450 bp, >40% of the expressed DUX4-S proteins were dimers or higher order multimers produced from trans-spliced mRNAs. As cDNA lengths increased, however, there was a decrease in the extent of dimer and multimer expression, indicating that trans-splicing became less efficient as the distance between the upstream acceptor and downstream donor sites was increased. For comparison, when using PTMs with an engineered binding domain, different groups have generated trans-spliced mRNAs with efficiencies of ~5–40%^14^.

Trans-splicing in the plasmid system was able to occur between RNAs transcribed from different plasmids, as demonstrated by our finding that heterodimers were generated between DUX4-S and either DUX4-FL or dok7. With the strong simian CMV IE94 promoter used here, multiple transcripts may be sequentially initiated and at different stages of synthesis from a single DNA at any one time, and such transcripts could have closely apposed splice donor and acceptor sites. Though further work is necessary to determine if trans-splicing may occur between such concurrently transcribed RNAs, it is clear that RNAs produced from different plasmids can be trans-spliced. Additional work, perhaps with spliceosome inhibitors^28^, is needed to determine if trans-splicing uses the same molecular mechanisms as cis-splicing.

Perhaps upstream trans-splicing will prove to share mechanisms with the still incompletely understood upstream “back-splicing” that generates circular RNAs from a single precursor transcript^29,30^. However, back-splicing by itself could not have generated the multimeric mRNAs we identified because trans-splicing between separate RNAs would have had to occur before any back-splicing to form circular RNA occurred within a single mRNA. In addition, our experiments showing that heterodimeric mRNAs (and proteins) formed when two plasmids were co-transfected provides definitive evidence that trans-splicing occurs. The co-transfection experiments also eliminated the possibility that chimeric mRNAs had arisen from plasmid dimers (or multimers) formed during bacterial amplification, a conclusion also supported by agarose gel analyses of the plasmids which showed no significant formation of plasmid dimers (Supplementary Fig. S6).

The co-transfection experiment with *dok7* and *DUX4-s* showed that trans-splicing could also generate both dok7/DUX4-S and DUX4-S/dok7 heterodimers. Thus, the different sizes of the dok7 (~1900 nucleotides) and DUX4-S coding sequence (~480 nucleotides) did not appear to promote trans-splicing in an exclusive orientation. However, by mutating only the acceptor site on one plasmid and the donor site on the second plasmid, it would be possible to generate heterodimers exclusively in only one of the two possible orientations.

This plasmid-based trans-splicing system presents new opportunities for investigating molecular mechanisms of trans-splicing, generating within cells covalent protein multimers with novel properties, and producing large proteins from split precursors. For example, it might be useful to incorporate features of the plasmid system into AAV vectors as a way to use mRNA trans-splicing, rather than concatemerization^31,32,33^, as the basis of a multiple virus strategy for expression of very large proteins such as dystrophin, nebulin, or titin which are mutated in muscle diseases^34^. Trans-splicing could also be used to test hypotheses about the possible functions of homo- and hetero-multimeric proteins vs. monomers. One example would be to determine if the multimer of a usually monomeric transcription factor is more effective in activating particular genes, perhaps indicating cooperative binding to multiple promoter binding sites. By adding an inducible promoter, it would be possible to modify a target protein, e.g. by adding a new functional domain or replacing a mutated domain, under controlled conditions for analysis of effects within the host cell. Whether this system might be useful for targeting endogenous, perhaps particularly abundant, mRNAs remains to be determined. However, the system does provide an example of efficient upstream trans-splicing in the absence of a PTM with an engineered binding domain, thus identification of the mechanism(s) underlying trans-splicing vs. cis-splicing in this system could identify methods to promote (or inhibit) particular patterns of splicing in other settings.

In summary, we identified a plasmid-based system in which mRNA trans-splicing occurred via joining a downstream splice donor to an upstream splice acceptor. This process of upstream trans-splicing generated chimeric mRNAs that encoded covalent, head-to-tail protein multimers. Dimers were the most abundant multimer, but higher order multimers (trimers, tetramers) were also found. Our study provides a cautionary example of the potential for unexpected upstream trans-splicing events in mRNAs generated from a commonly used plasmid and epitope tag. On the other hand, this trans-splicing system should be adaptable to multiple gene delivery methods and it also presents new opportunities for investigating molecular mechanisms of trans-splicing, identifying novel functions of covalent protein multimers, and generating larger proteins from split precursors.

## Materials and Methods

### Plasmids

The pCS2(+)-V5 host vector and the different DUX4 constructs, as listed in Fig. 1B, were prepared as described previously^19,21^. The NCBI reference sequence for the full-length DUX4 protein is NP_001292997.1; and the sequence of pCS2(+) plasmid (without the V5 epitope) is available online at https://www.addgene.org/vector-database/2295/. The V5 epitope sequence used in the constructs listed in Fig. 1B contained two potential splice donor sites as noted by Ansseau *et al*.^23^ and discussed further below. However, the 3′ regions of our DUX4 constructs used the SV40 (polyA) sequence which Ansseau *et al*.^23^ predicted would not generate aberrant downstream splicing.

**Table 1:** PCR primers used in this study.

Number	Name	Sequence
Primer 01	EGFP-Fw-XhoI	ACCGCTCGAGATGGTGAGCAAGGGCGAGGAG
Primer 02	EGFP-Rv-XbaI	TGTCTAGATTACTTGTACAGCTCGTCCATGCC
Primer 45	z-dok7–201-Fw-EcoRI	GAATTCCACAAGATGACGGATACGGTTGTC
Primer 46	z-dok7–201-nonstop-Rv-XhoI	CTCGAGTGTGAGTGTTCCTCTCCTCTTGTG
Primer 51	XhoI-KpnI-FLAG-XbaI-Fw	TCGAGGGTACCCGGTTCGAAGACTACAAAGACGATGACGACAAGT
Primer 52	XhoI-KpnI-FLAG-XbaI-Rv	CTAGACTTGTCGTCATCGTCTTTGTAGTCTTCGAACCGGGTACCC
Primer 269	RT-PCR-DUX4-3UTR-Rv	ATGTCTGGATCTACGTAATACGACTCAC
Primer 270	RT-PCR-DUX4s-Fw	AGTCCAGGATTCAGATCTGGTTTC
Primer 271	RT-PCR-DUX4s-Rv	ACCTCTCATTCTGAAACCAAATCTG
Primer 309	RT-PCR-z-dok7-Rv	CGAAATGCCACGAACGATGCAATCCA
Primer 310	RT-PCR-z-dok7-Fw	CCTGACTGTGGCGGACGAAAGGTG

Plasmids for expression of HA epitope-tagged DUX4-FL and Myc epitope-tagged DUX4-S were generated and provided by Dr. Peter L. Jones (University of Nevada School of Medicine at Reno). The host plasmid for these constructs was pcDNA3.1, and the epitope tags were at the N-terminus of the expressed protein.

To generate a plasmid for expression of a DUX4-S-EGFP fusion protein, the EGFP coding sequence was amplified by PCR with primers 01 and 02 (Table 1) and inserted into XhoI- and XbaI-sites in the pCS2(+) vector to obtain pCS2(+)-EGFP. Then the DUX4-s-pCS2(+)-V5 plasmid was digested with EcoRI and XhoI, and the *DUX4-s* coding sequence was inserted into the EcoRI and XhoI sites of *pCS2(*+*)-EGFP*. The resulting *DUX4-s-EGFP* plasmid encoded DUX4-S fused to EGFP at the C-terminus.

To obtain FLAG-tagged DUX4 constructs, primers 51 and 52 (Table 1) were annealed and inserted into XhoI and XbaI sites of the pCS2(+) vector to generate the FLAG epitope coding sequence. The *DUX4-fl* or *DUX4-s* coding sequence was then inserted into the EcoRI and XhoI sites of the resulting vector so that DUX4-FL or DUX4-S would be expressed with the FLAG epitope tag at the C-terminus.

To generate the pCS2(+)-*dok7*-V5 plasmid, the coding sequence of zebrafish *dok7* was amplified by PCR with primers 45 and 46 (Table 1) using cDNA from zebrafish line RIKEN WT (ZFIN ID: ZDB-GENO-070802–4). The PCR product was cloned into the pGEM-T-easy vector (cat.A1360, Promega, Madison WI). Though differing a nine nucleotides, Sanger sequencing confirmed that the cloned zebrafish *dok7* cDNA encoded a protein with the same amino acid sequence as the NCBI reference sequence XM_681035.1^35^. The cloned *dok7* sequence was digested with EcoRI and XhoI and then inserted into the pCS2(+)-V5 host plasmid.

### Cells and culture

Cells of the human embryonic kidney line 293 (HEK293) were obtained from the American Type Culture Collection, Manassas VA (cat. CRL1573) and HeLa cells were obtained from the RIKEN BRC Cell Bank (cat. RCB0007, Tsukuba, Japan). Cells were grown in Eagle’s Minimum Essential Medium (cat. 30–2003, American Type Culture Collection, Manassas VA, USA) or Dulbecco’s Modified Eagle’s Medium (cat. D5796, Sigma-Aldrich) supplemented with 10% fetal bovine serum (cat. 10270-106, Thermo-Fisher, Grand Island NY; or cat. SH30070, HyClone GE Life Sciences, Logan UT).

### Transfection

Plasmids were transfected into HEK293 or HeLa cells using the X-treme GENE HP DNA transfection reagent (cat. 6366244001, Sigma-Aldrich) diluted in Opti-MEM I (Gibco) following the manufacturer’s instructions. For pCS2(+)-derived plasmids, expression was under control of the simian CMV IE94 promoter fragment. The percentage of host cells that expressed each construct, typically >90%, was monitored by immunofluorescence as described^19^.

### BacMam vectors

BacMam vectors used to express DUX4-FL and DUX4-S under control of a human CMV-IE1 promoter were as described previously^36–39^. Viral supernatants were used without further purification and were added to cell cultures at a level that generated expression in >90% of host cells at 48 h after addition of the virus.

### Antibodies

Rabbit anti-DUX4 mAb E55 which reacts with a C-terminal domain epitope of the full-length (424aa) protein^22^ was used at 1:200 dilution (cat. ab124699, Abcam, Cambridge MA). GFP was detected with mouse mAb 4B10 (cat. 2955, Cell Signaling Technology) used at 1:500 dilution. GAPDH was detected with a mouse mAb (cat. 10R-G109A, Fitzgerald, Acton MA) used at 1:5000 dilution. The V5 epitope tag was detected using either mouse anti-V5 mAb (cat. R960-25, Thermo Fisher) used at 1:500 or a rabbit pAb (cat. AB3792, EMD Millipore) used at 1:300. The FLAG epitope tag was detected with anti-FLAG mAb M2 (cat. F1804, Sigma-Aldrich) used at 1:5000 dilution. The Myc epitope tag was detected with a mouse mAb (cat. 2276, Cell Signaling Technology) used at 1:500 dilution. Each of the primary antibodies was validated based on one or more methods, including prior use in multiple published studies with the same mAb or lot of polyclonal antiserum, manufacturer’s validation assays including knockouts, generation of expected immunofluorescence staining patterns, detection of appropriate band size on immunoblots without detection of non-specific bands, and detection of recombinant protein when expressed in cells that normally do not express the protein.

### Immunoblots

Immunoblotting was performed as described previously^35^. Immunoblots were quantified using the grey scale densitometric function of the NIH ImageJ software v.1.51 available at https://imagej.nih.gov/ij/download.html. Immunoblots presented in the figures are representative of experiments that had been independently repeated two or more times.

### RT-PCR, primers, and sequencing

At 24 h after transfection, cells were harvested and total RNA was extracted with TRIzol reagent (cat. 15596026, ThermoFisher) followed by affinity purification by RNeasy column (cat. 74104, QIAGEN) with DNase I treatment (Sigma-Aldrich). The procedure for cDNA synthesis was as described previously^19^. Transcripts generated by trans-splicing were amplified by PCR with PrimeSTAR GXL DNA polymerase (cat. R050A, Takara) with the following cycling conditions: 98°C 2 min followed by 30 cycles of 98°C for 10 sec and 68°C for 1 min. The locations of the primers are diagramed in Fig. 3 and Fig. 8; and the primer sequences are listed in Table 1. PCR products were electrophoresed and visualized with LAS-3000 (FUJIFILM). All RT-PCR products were gel-purified, cloned into the PCR blunt vector (cat. K275020, ThermoFisher), and sequenced in both directions. Sanger sequencing of DNA constructs and RT-PCR products was performed by the Support Center for Medical Research and Education, Tokai University using an ABI 3500xL Genetic Analyzer (Applied Biosystems, Foster City, CA, USA).

### Splice site prediction and plasmid mutagenesis

We first used the online implementation of the NetGene2 splice site prediction program^38,39^ http://www.cbs.dtu.dk/services/NetGene2/) to identify likely splice acceptor and donor sites in possible mRNAs transcribed from the pCS2(+)-DUX4-S-V5 plasmid. The plasmid sequence was analyzed as a circular form with human sequence. For each potential acceptor or donor site, the program returned a confidence value. The prediction program classified confidence values ≥0.5 as “nearly all true” for donor sites whereas the “nearly all true” confidence values for acceptor sites were ≥ 0.2. As noted in the text, the acceptor 1, donor 1, and donor 2 sites identified in our work all carried confidence values in these “nearly all true” categories. In contrast, the mutagenized acceptor 1, donor 1, and donor 2 sites (Fig. 5) were not predicted to be functional splicing sites. Plasmid mutagenesis was carried out by a commercial service (GeneWiz, South Plainfield NJ), and all mutagenized plasmids were verified by re-sequencing. As described in Supplemental Data, additional splice site analyses were carried out with the Human Splice Finder tool (HSF3.1, accessed at http://www.umd.be/HSF3/index.html) and the Berkeley Drosophila Genome Project tool configured for human splice sites (accessed at http://www.fruitfly.org/seq_tools/splice.html).

## Electronic supplementary material

Mitsuhashi et al. Supplementary Information
