# Supplementary material for: Efficient system for upstream mRNA trans-splicing to generate covalent, head-to-tail, protein multimers
Source: Sci Rep. 2019 Feb 19;9:2274. doi: 10.1038/s41598-018-36684-7 (PMC6381186; doi:10.1038/s41598-018-36684-7)

SUPPLEMENTARY RESULTS AND FIGURES.

Efficient system for upstream mRNA trans-splicing to generate covalent, head-to-tail, protein multimers

Hiroaki Mitsuhashi[1#], Sachiko Homma[2], Mary Lou Beermann[2], Satoshi Ishimaru[1], Hayato Takeda[1], Bryant K. Yu[2], Kevin Liu[2], Swetha Duraiswamy[2], Frederick M. Boyce[3] and Jeffrey Boone Miller[2#]

[1]Department of Applied Biochemistry
School of Engineering
Tokai University
Kanagawa 259-1207, Japan

[2]Department of Neurology
Boston University School of Medicine
Boston, Massachusetts USA 02118

[3]Department of Neurology
Massachusetts General Hospital
Boston, Massachusetts USA 02114

SUPPLEMENTARY RESULTS

As shown in Fig. 2 of the main text, apparent protein multimers were generated when the pCS2(+)-DUX4-V5 plasmids were transfected into HeLa or HEK293 cells. In the supplementary text and figures, we describe how we characterized these multimers as covalent proteins and identified key aspects of the plasmids that were required for multimer formation.

Because the potential multimers were stable upon boiling for 5 min. in our SDS sample buffer, we determined if alterations to sample preparation might eliminate the possible multimers. In particular, we tested different temperatures and lengths of heating, different concentrations and lots of dithiothreitol and beta-

mercaptoethanol, and solubilizing the transfected cells in different concentrations of urea. However, none of these treatments affected the banding pattern of proteins expressed from the transfected pCS2(+)-V5 plasmids (not shown). We also found that dimers continued to be formed when the amount of *DUX4-s* plasmid used for transfection (normally 5 µg) was decreased to 0.5 µg or 0.05 µg by 1:10 or 1:100 dilution with the empty control vector pCS2(+)-V5 (not shown).

We next carried out a series of experiments to determine if formation of the potential multimers was associated with the V5 epitope tag in the pCS2(+)-V5 plasmid. We first found that DUX4-S fused at its C-terminus with EGFP (DUX4-S-EGFP), which was expressed from pCS2(+), did not appear to form dimers, because the largest band we found was at ~40kDa as expected for the monomer of this construct (Fig. S1A). Additional smaller bands (e.g., ~20kDa and ~25kDa) that reacted with the anti-GFP antibody could have been due to incomplete translation or proteolysis. Similarly, we found no evidence of dimer formation by Myc-DUX4-S, expressed from pcDNA3.1 (Fig. S1B) or by DUX4-S-FLAG expressed from pCS2(+) (not shown). Next, we carried out single transfections of DUX4-FL-V5 or HA-DUX4-FL, as well as co-transfection of DUX4-FL-V5 and HA-DUX4-FL to search for interactions (Fig. S1C). In this experiment, we found that apparent dimers were formed by DUX4-FL-V5 in both single and co-transfections, but dimers were not formed by the HA-DUX4-FL in either single or co-transfections. The amount of DUX4-FL-V5 dimer was reduced in the co-transfection due, at least in part, to the use of one-half the amount of DUX4-FL-

V5 plasmid in the co-transfection compared to the single transfection. Taken together, the results in Fig. S1 suggested that formation of the apparent dimers required that the host plasmid include the V5 epitope as found in the pCS2(+)-V5 host plasmid.

We then determined if formation of the apparent dimers required expression of the constructs within a cell. When we carried out coupled *in vitro* transcription and translation of the DUX4-FL-V5, delMid-V5, and DUX4-S-V5 plasmids, we found that the largest V5-containing products were of the sizes expected for the monomers of each construct (i.e., ~50kDa, ~32kDa, and ~20kDa respectively) (Fig. S2A); thus, we found no evidence that SDS-stable multimers were formed. This result confirmed that the plasmids did not contain multiple head-to-tail copies of cDNAs that could have generated a multimeric fused transcript and protein (a result also confirmed by our sequencing of each plasmid). In addition, this experiment showed that events within a host cell were likely required for formation of the apparent multimers.

With the understanding that the formation of the apparent dimers depended both on the V5 epitope and expression within a cell, we next asked if the host expression vehicle affected multimer formation. For this work, we expressed DUX4-FL-V5 and DUX4-S-V5 either from a BacMam virus (Fig. S2B), as in our previous work (Homma et al., 2015, 2016), or by transfection of the plasmid that was used to generate the BacMam virus (Fig. S2C). Compared to pCS2(+)-V5, the BacMam system has a different 5' sequence upstream of the DUX4 cDNA that includes a human CMV-IE1 promoter and intron, as well as a

different 3’UTR that includes an IRES-GFP expression cassette (Homma et al., 2015, Fornwald et al., 2016, Mansouri et al., 2016). When HEK293 cells were either infected with the BacMam virus (Fig. S2B) or transfected with the BacMam plasmid (Fig. S2C), we found that the largest V5-tagged bands were of the sizes expected for DUX4-FL and DUX4-S monomers, and there were no bands of the sizes expected for dimers or higher order multimers. Parallel transfections with the corresponding pCS2(+)-DUX4-FL-V5 and pCS2(+)-DUX4-S-V5 plasmids, in contrast, generated bands of the sizes expected for apparent dimers (and trimers in the case of DUX4-S) in addition to the band of the sizes expected for monomers. These results suggested that features specific to the pCS2(+)-V5 host plasmid, in addition to the presence of the V5 epitope sequence, determined whether apparent dimers could be generated.

Also, we used additonal online prediction tools to analyze splice site strength and to look for the presence of splicing enhancers in the DUX4-S-V5 and dok7-V5 mRNAs transcribed from the pCS2(+) vector. We analyzed the two different expression vectors to control for possible effects of different coding sequences. Using the Human Splice Finder tool (HSF3.1, accessed at http://www.umd.be/HSF3/index.html), we found that the acceptor (A1) and donor (D1, D2) splice sites were predicted to be the strongest splice sites in both the DUX4-S-V5 and dok7-V5 mRNAs (Supplemental Fig. S3). The acceptor and donor sites that we identified in the Ampicillin resistance gene were also predicted to be strong sites (Supplemental Fig. S3B). In addition, a complex landscape of possible splicing enhancers and silencers was predicted in the

DUX4-S-V5 mRNA (Supplemental Fig. S4). However, the signficance of these many possible splice site regulators remains to be determined. Splice site enhancers can affect sites that are several hundred nucleotides away (Tian and Maniatis, 1994), and further analyses of the many possible splicing regulatory regions was beyond the scope of this project.

SUPPLEMENTARY REFERENCES

Fornwald, J. A, Lu, Q., Boyce, F. M and Ames, R. S. (2016). Gene Expression in mammalian cells using BacMam, a modified baculovirus system. *Methods Mol. Biol*. **1350**, 95-116.

Homma, S., Beermann, M. L., Boyce, F. M. and Miller, J. B. (2015). Expression of FSHD-related DUX4-FL alters proteostasis and induces TDP-43 aggregation. *Ann. Clin. Transl. Neurol*. **2**, 151-166.

Homma, S., Beermann, M. L., Yu, B., Boyce, F. M. and Miller, J. B. (2016). Nuclear bodies reorganize during myogenesis in vitro and are differentially disrupted by expression of FSHD associated DUX4. *Skelet. Muscle* **6**(1):42.

Mansouri, M., Bellon-Echeverria, I., Rizk, A., Ehsaei, Z., Cianciolo Cosentino, C., Silva, C. S., Xie, Y., Boyce, F. M., Davis, M. W., Neuhauss, S. C., Taylor, V., Ballmer-Hofer, K., Berger, I. and Berger, P. (2016). Highly efficient baculovirus-mediated multigene delivery in primary cells. *Nat. Commun*. **7**, 11529.

Tian M, Maniatis T. (1994) A splicing enhancer exhibits both constitutive and regulated activities. *Genes Dev*. **8**, 1703-1712.

SUPPLEMENTARY FIGURE LEGENDS

Fig. S1. Only V5-tagged DUX4 proteins cloned in the pCS2(+)-V5 formed SDS-resistant apparent multimers. **A.** A DUX4-S-EGFP fusion protein did not form SDS-resistant multimers. At 48h after transfection of 5 µg or 0.5 µg of DUX4-s-EGFP plasmid in HEK293 cells, the largest band detected by immunoblotting with anti-GFP was at the size (~42kDa) expected for the DUX4-S-EGFP monomer. No bands of the sizes expected for apparent dimers or higher order multimers were detected. Smaller bands could be incomplete translation or proteolysis products. **B.** A Myc-DUX4-S protein expressed from a pcDNA3.1 host plasmid did not form SDS-resistant multimers. Expression of DUX4-S-V5 vs. Myc-DUX4-S epitope tagged proteins was analyzed by immunoblotting 48h after transfection of 5 µg plasmid in HEK293 cells. The V5-tagged protein, but not the Myc-tagged protein, showed bands of the sizes expected for a dimer and (faintly) a trimer. **C.** A HA-DUX4-FL protein did not form SDS-resistant multimers. As indicated, HEK293 cells were either mock-transfected (None); co-transfected with 2.5 µg each of HA-DUX4-FL and DUX4-FL-V5 (co-transfect HA & V5); transfected only with 5 µg HA-DUX4-FL (HA only); or transfected only with 5 µg DUX4-FL-V5 (V5 only). Duplicate immunoblots were analyzed with anti-HA (left) or anti-V5 (right) at 48h after transfection. Bands the size of apparent dimers were produced by DUX4-FL-V5, but not HA-DUX4-FL. The amount of apparent DUX4-FL-V5 dimer was reduced in the co-transfection lane consistent with half as much V5 plasmid being used in the transfection.

Fig. S2. V5 epitope-tagged DUX4 proteins did not form multimers when generated by *in vitro* synthesis or when expressed from either a BacMam virus vector or the plasmid used to generate the BacMam virus. **A.** DUX4-FL-V5, delMid-V5, and DUX4-S-V5 proteins were produced by coupled *in vitro* transcription and translation of the corresponding pCS2(+)-V5 plasmids described in Fig. 1. For each construct, SDS-PAGE and anti-V5 immunoblotting detected a band of the size expected for the monomer, but no larger bands of the sizes expected for dimers or higher order multimers were detected. **B, C.** DUX4-FL-V5 and DUX4-S-V5 were expressed in HEK293 cells either by infection with a BacMam virus vector (**B**) or by transfection of the pCS2(+) plasmid construct (**C**). The BacMam virus carried the same cDNA, including the linker and V5 tag, as the corresponding pCS2(+)-V5 plasmid, but the BacMam vector had different 5' and 3' UTRs than the pCS2(+)-V5 plasmid. After 48h, the expressed proteins were examined by SDS-PAGE and anti-V5 immunoblotting. As in previous figures, DUX4-FL-V5 and DUX4-S-V5 expressed from the pCS2(+)-V5 plasmid showed SDS-resistant bands of the sizes expected for dimers and, for DUX4-S-V5, higher order multimers. When expressed from the BacMam virus vector or BacMam plasmid, in contrast, only bands of the sizes expected for monomers were found.

Fig. S3. Splice site strength prediction. **A.** We used the Human Splice Finder tool HSF3.1 (accessed at http://www.umd.be/HSF3/index.html) to analyze 975 nucleotides of the pCS2(+)-DUX4-s-V5 sequence from the transcription start site (TSS) through the SV40 poly(A) site. The upper diagram shows regions of

the transcript, including the Acceptor 1 (A1), Donor 1 (D1), and Donor 2 (D2) characterized in Fig. 5A. The lower graph shows possible acceptor sites (light blue squares) and donor sites (magenta circles) with the predicted strength of the site graphed on a scale of 0 (none) to 100 (strongest). The A1 site was the strongest predicted acceptor site and the D1 and D2 sites were the two highest scoring donor sites. **B.** Numerical predicted strengths of splice sites. We used both the HSF3.1 tool and the Berkeley Drosophila Genome Project (Berkeley) tool configured for human splice sites (accessed at http://www.fruitfly.org/seq_tools/splice.html) to analyze splice site strengths of the Acceptor 1, Donor 1, and Donor 2 sites of the pCS2(+)-DUX4-s-V5 and pCS2(+)-dok7-s-V5 sequences. The predicted strengths were independent of the cDNA. In addition, we analyzed splice site strengths for the acceptor (*AmpR* Acceptor) and donor (*AmpR* donor) sites that we identified in the reverse orientation ampillin resistance sequence (Fig. 4C, 4D).

Fig. S4. Prediction of splice site enhancers (upper part of graph) and silencers (lower part of graph) in the DUX4-S-V5 mRNA by the Human Splice Finder tool HSF3.1 with a scale of 0 – 100. The tool predicted a complex landscape of splicing regulators in the sequences surrounding the A1, D1, and D2 sites which are indicated on the uppermost diagram. A complete description of the analytical tool can be found at: http://www.umd.be/HSF3/index.html).

Fig. S5. Complementary sequence analysis of the upstream region (labeled A = 111 nucleotides) vs. the downstream region (labeled B = 260 nucleotides) of the DUX4-S-V5 mRNA. The two highest scoring outcomes of the

analysis using a two sequence complementarity tool (accessed at https://tinyurl.com/twosequenceanalyzer) are shown. The longest perfect matches were only four nucleotides and the greatest number of matches in a fifteen nucleotide sequence was ten. In contrast, as noted in the text, engineered pre-trans-splicing molecules use binding domains with perfectly complementary sequences that are typically ≥50 nucleotides long.

Fig. S6. Plasmid dimers were not found in plasmids used for transfection in this study. Shown is an agarose gel with 250 ng of the indicated plasmid analyzed in each lane. As indicated, each preparation included the bands of the electrophoretic mobilities exprected for supercoiled and open coiled forms of the plasmid monomers. In contrast, the analysis showed no significant bands of larger size that could have been plasmid dimers.

Fig. S1. Mitsuhashi et al.

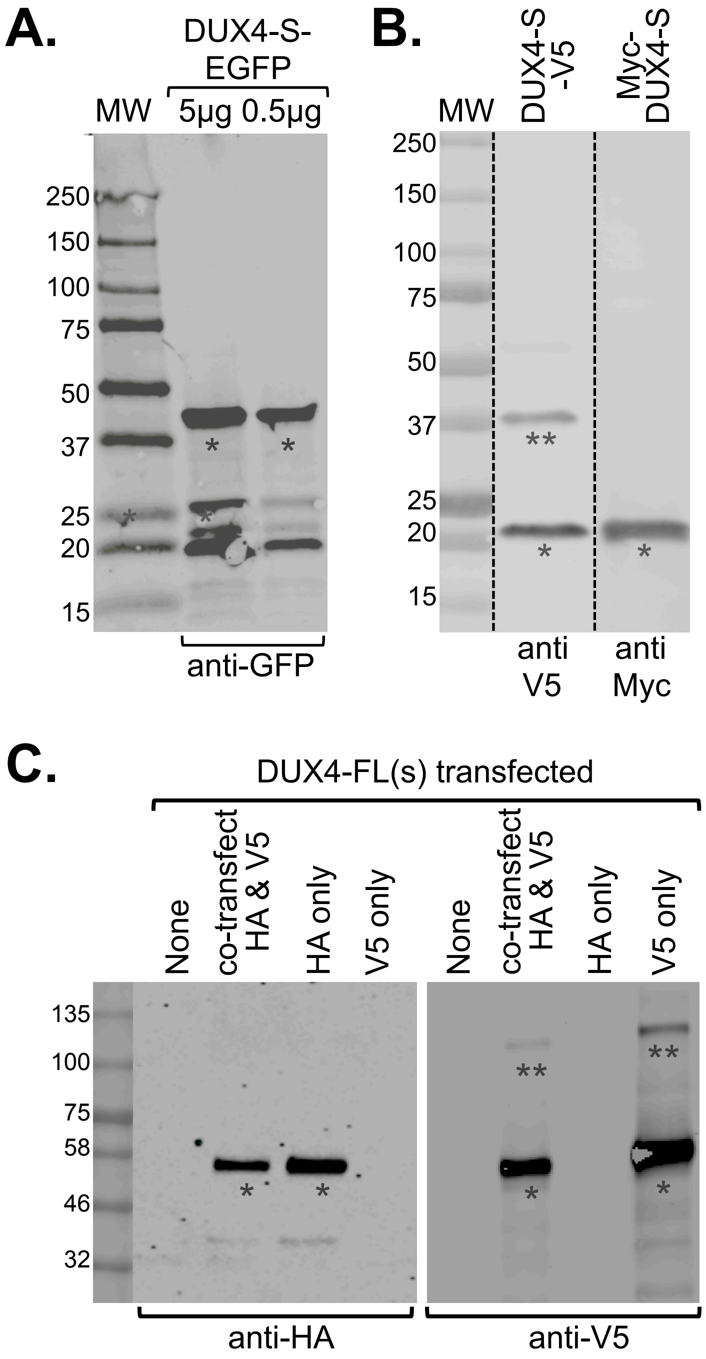

Fig. S2. Mitsuhashi et al.

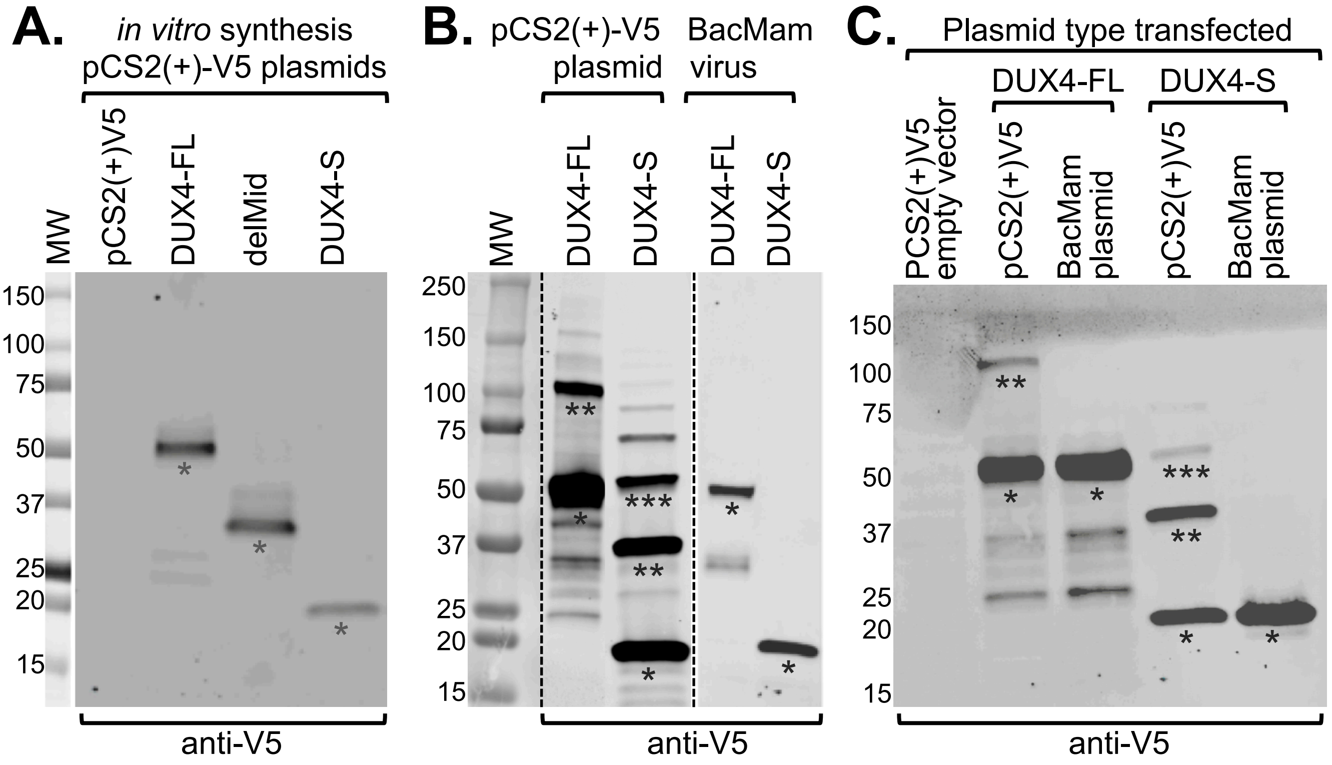

Fig. S3. Mitsuhashi et al.

A.

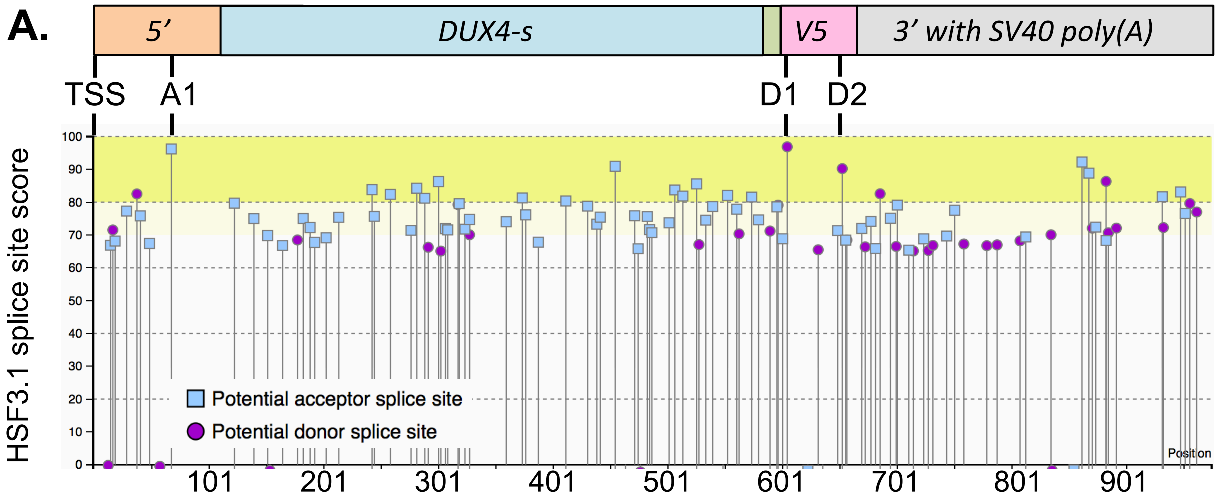

B.

Splice Site	Plasmid	HSF3.1 score*	Berkeley score**
Acceptor 1	*DUX4-s-V5*	96.22	0.99
	dok7-V5	96.22	0.99
Donor 1	*DUX4-s-V5*	96.87	1.00
	dok7-V5	96.87	1.00
Donor 2	*DUX4-s-V5*	90.23	0.99
	dok7-V5	90.23	0.99
AmpR Acceptor	*DUX4-s-V5*	79.21	0.63
AmpR Donor	*DUX4-s-V5*	83.61	0.98

*Splice site strength score using Human Splice Finder 3.1 tool with a scale of 0 (none) – 100 (strongest).

**Splice site strength score using Berkeley Drosophila Genome Project tool with a scale of 0 (none) – 1.0 (strongest).

Fig. S4. Mitsuhashi et al.

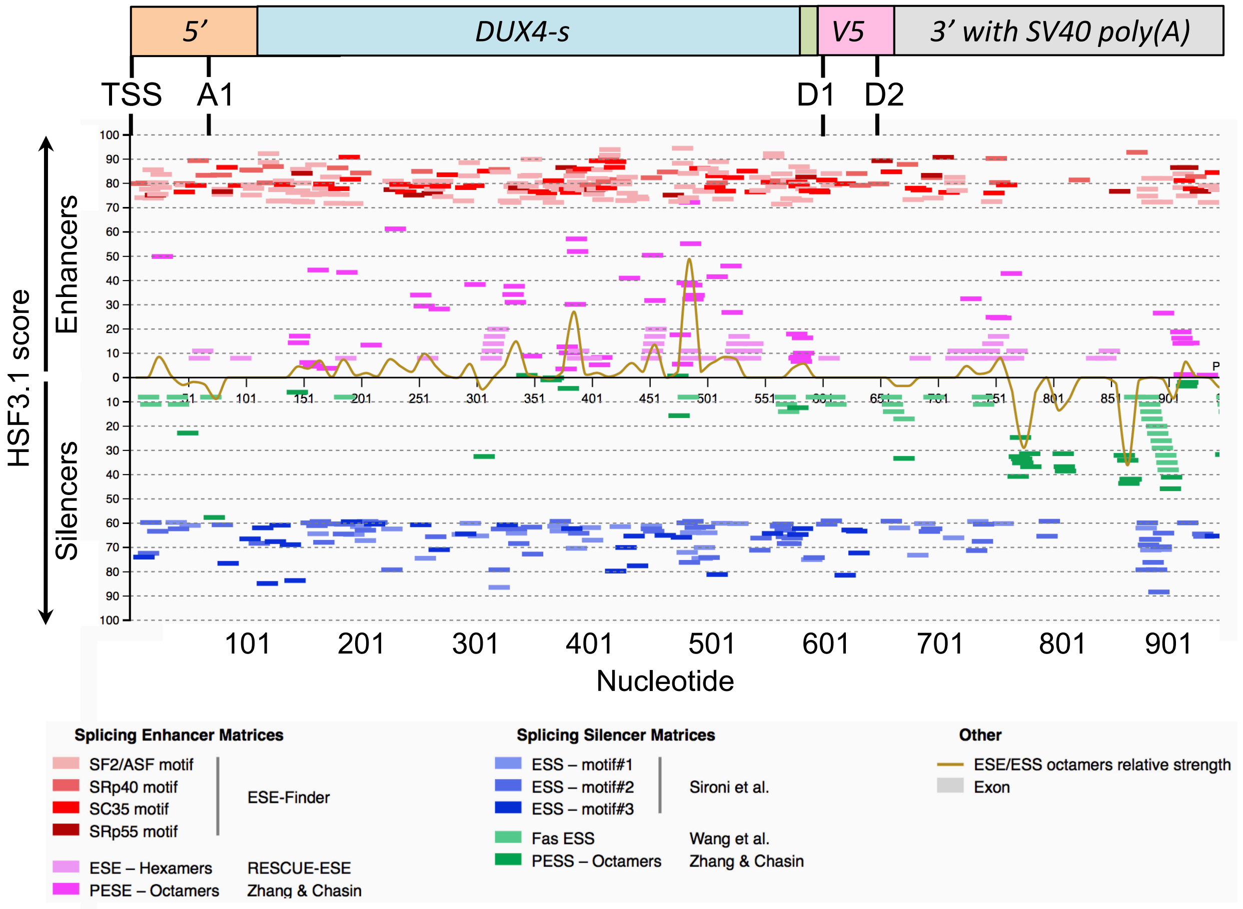

Fig. S5. Mitsuhashi et al.

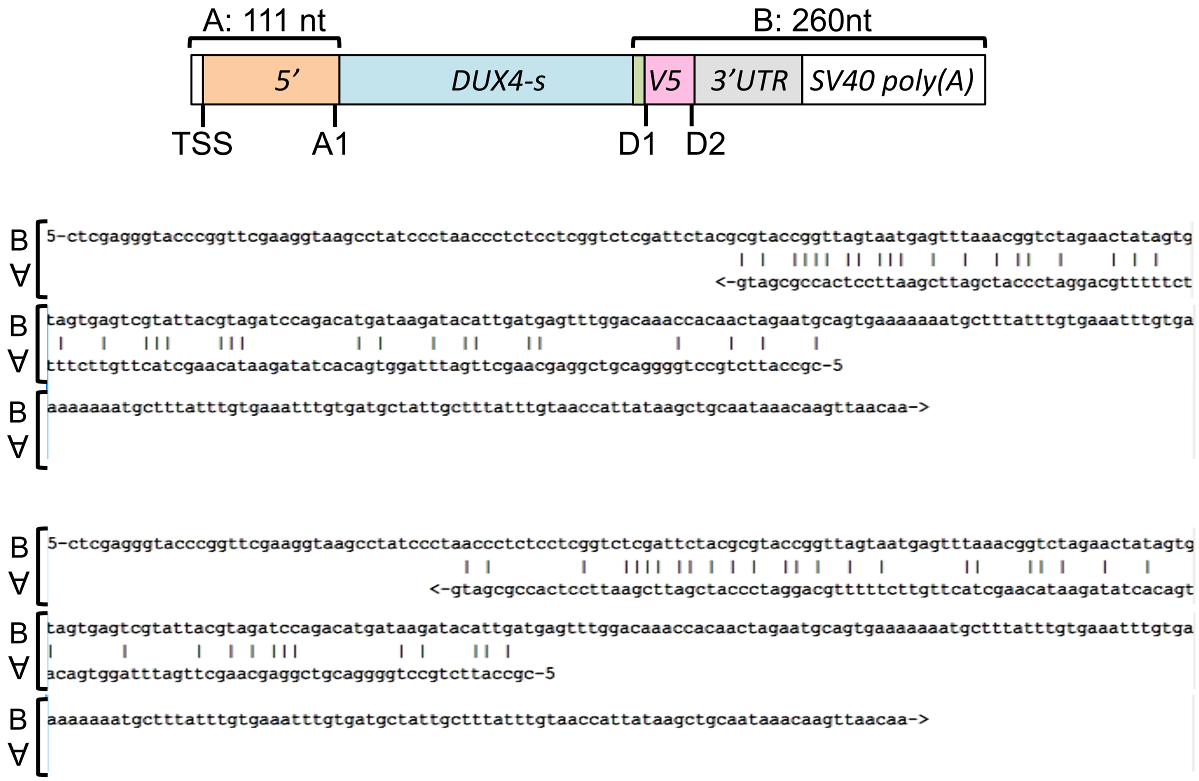

Fig. S6. Mitsuhashi et al.

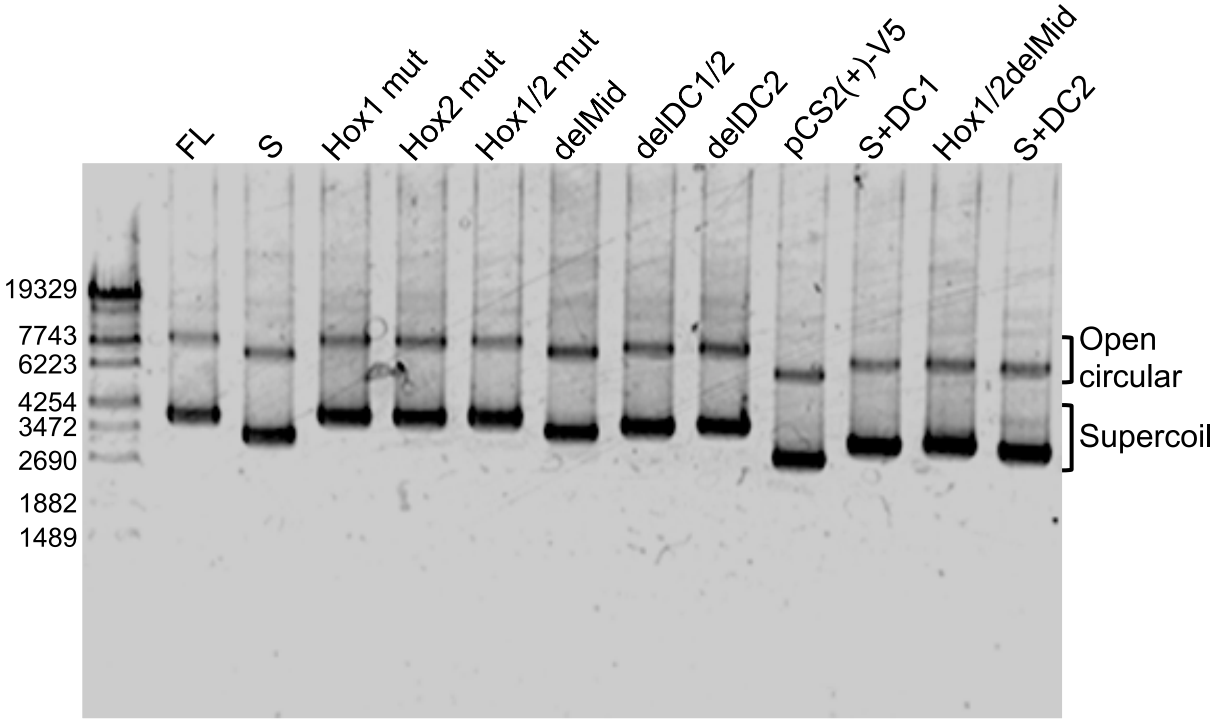

Supplement: Supplementary file 1 — Mitsuhashi et al. Supplementary Information [file 41598_2018_36684_MOESM1_ESM.pdf]
